# Supplementary material for: Young-parent communication on sexual and reproductive health issues among young female night students in Amhara region, Ethiopia: Community-based cross-sectional study
Source: PLoS One. 2021 Jun 18;16(6):e0253271. doi: 10.1371/journal.pone.0253271 (PMC8213161; doi:10.1371/journal.pone.0253271)
Supplement: S1 File — (DOCX) [file pone.0253271.s001.docx]

**Debre Tabor University College of health sciences Department of Midwifery**

**English Version Questionnaire**

**Consent form**

This questionnaire develops to assess Young-Parent Communication on Sexual and Reproductive Health Issues among Young Female Night Students in Amhara Region.
Good morning/afternoon, my name is-----------------------. I am working as a data collector for the study being conducted in this community by Gedefaye Nibret and his colleagues who are doing a research in Debre Tabor University, College of Health Sciences. The purpose of this questionnaire is to gather information to assess of young-parent communication on sexual and reproductive health issues among young female night students in Amhara Region. We have identified you as a study participant hoping that you would be willing to help me by providing with some information. I have several questions which I would like to ask you, if you have the time and are willing. The questionnaires include socio demographic factors, reproductive health characteristics and communication about reproductive health issues related questions. Honest answers would contribute to identify the gaps. Your role in the success of the research is important and I appreciate your contribution to the research. Would this be okay with you? I understood about the advantage of the research and agreed to Participate in the research.

A. Yes, ------------sign.

B. No, thank you

| Questionnaire Code |  |
| --- | --- |
| Date of data collection |  |
| Name of supervisor |  |
| Name of data collector |  |
| Site of data collection |  |

If you have any question you can contact at any time with the following address.

Name: Gedefaye Nibret

Tele: +251 918159217

E-mail: [gedefayen@gmail.com](mailto:gedefayen@gmail.com)

**Part I. Socio-demographic characteristics of adolescent respondents and their parents (encircle your choice)**

| S/No | Question | Response | Skip |
| --- | --- | --- | --- |
| 101 | How old are you? | ___________years |  |
| 102 | Marital status | 1. Married  2. Had boy friend  3. Had no partner |  |
| 103 | What is your religion | 1.Orthodox  2.Muslim  3.Catholic  4.Protestant  5.if Other Specify----------------- |  |
| 104 | What is your ethnicity | 1.Amhara  2.Oromo  3.Tigre  4. others, specify----------------- |  |
| 105 | Area of residence? | 1.Urban  2.Rural |  |
| 106 | What is Your occupation? | 1.House wife  2. Daily laborer  3. Merchant  4. Government employee  5. Maid  6. commercial sex worker  7. if other specify------------- |  |
| 107 | Level of education in grades | -------------grade |  |
| 108 | Mother’s educational status | 1. Unable to write and read  2.Read and write only  3.Primary education(1-4)  4.Primary education(5-8)  5.Secondary education(9-12)  6.College and above |  |
| 109 | Father’s educational status | 1.Unable to write and read  2.Read and write only  3.Primary education(1-4)  4.Primary education(5-8)  5.Secondary education(9-12)  6.College and above |  |
| 110 | Mother’s current occupational status | 1.House wife  2.Market vendor/trader  3.Daily laborer  4.Government employee  5.Others(Specify) |  |
| 111 | Father’s current occupational status | 1.farmer  2. Merchant  3.Daily laborer  4.Civil servant  5.Others(Specify) |  |
| 112 | With whom do you live now? | 1. live alone  2.with both parents  3.only with my father  4.only with my mother  5.with relatives  6.with sexual partner  7.with my friend  8. with employeer  9. others(specify) |  |
| 113 | Are you currently having your own income? | 1. Yes  2. No |  |
| 114 | How much is your monthly income? | -------------birr |  |

**Part II. Reproductive related characteristics of young female adolescents**

| 201 | Have you ever heard about sexual and reproductive health? | 1. Yes 2. No |  |
| --- | --- | --- | --- |
| 202 | What was your primary Source of information about reproductive health? | 1. School 2. Peer including girl or boy friend 3. Mass media (TV, Radio, Magazines, Newspaper) 4. Family (father and mother) 5. Brothers /sisters 6. Relatives 7. Other (specify) |  |
| 203 | Did you utilize youth friendly sexual and reproductive health services? | 1. Yes 2. No |  |
| 204 | Age at first marriage | ___________in years |  |
| 205 | Have you ever sexual intercourse? | 1. Yes 2. No |  |
| 206 | Did you face unwanted pregnancy? | 1. Yes 2. No |  |
| 207 | Did you face abortion? | 1. Yes 2. No |  |
| 208 | Did you face sexual transmitted infection before? | 1. Yes 2. No |  |
| 209 | Did you face sexual violence? | 1. Yes 2. No |  |
| 210 | If you say Qes. No 2009 What type of sexual violence’s | 1.rape  Unpleasant words |  |

**Part III. Parent adolescent communication concerning SRH issues.**

| 301 | Do you think it is important to discuss about sexual and reproductive health issues with parents? | 1. Yes 2. No |  |
| --- | --- | --- | --- |
| 302 | Have you ever discussed premarital sex with your parents? | 1. Yes 2. No |  |
| 303 | With whom do you discuss issues related to premarital sex | 1. Father 2. Mother 3. Brother or sister 4. Peer 5. Others(specify) |  |
| 304 | Have you ever discussed abortion with your parents? | 1. Yes 2. No |  |
| 305 | With whom do you discuss issues related to abortion? | 1. Father 2. Mother 3. Brother or sister 4. Peer 5. Others(specify) |  |
| 306 | Have you discussed contraception with your parents? | 1. Yes 2. No |  |
| 307 | With whom do you discuss issues related to contraception? | 1. Father 2. Mother 3. Brother or sister 4. Peer 5. Others(specify) |  |
| 308 | Have you discussed STI and HIV/AIDS within the past twelve months? | 1. Yes 2. No |  |
| 309 | With whom do you discuss issues related to STI and HIV/AIDS? | 1. Father 2. Mother 3. Brothers or Sisters 4. Peer 5. Others(specify) |  |
| 310 | Have you discussed about unwanted pregnancy with your parents? | 1. Yes 2. No |  |
| 311 | With whom do you discuss issues related to unintended pregnancy? | 1. Father 2. Mother 3. Brothers or sisters 4. Peer 5. Others(specify) |  |
| 312 | Have you about discussed about condom use with your parents? | 1. Yes 2. No |  |
| 313 | With whom do you discuss about early marriage? | 1. Father 2. Mother 3. Brothers or sisters 4. Peer 5. Others(specify) |  |

-------------------------------THANK YOU SO MUCH--------------------------
