# Supplementary material for: Young-parent communication on sexual and reproductive health issues among young female night students in Amhara region, Ethiopia: Community-based cross-sectional study
Source: PLoS One. 2021 Jun 18;16(6):e0253271. doi: 10.1371/journal.pone.0253271 (PMC8213161; doi:10.1371/journal.pone.0253271)
Supplement: Attachment — Submitted filename: reviwer comment for authors.docx [file pone.0253271.s002.docx]

Dear editor, I would like to say thank you for considering me to review the manuscript entitled as ‘**Young-Parent Communication on Sexual and Reproductive Health Issues among Young Female Night Students in Amhara Region, Ethiopia**

**General comment**

I have assessed your manuscript thoroughly and “I **am sorry to say that it might not be considered for publication in the present form**. I listed my comments below and I hope the comment would improve the manuscript and may allow a revised version to be reassessed otherwise not.

The scope of the study is not described clearly. Please clarify the detail and importance of **Young-Parent Communication on Sexual and Reproductive Health Issues under introductions**" Moreover the results of this study are somehow as expected, not new nor interesting, nor surprising: I do not believe this study adds anything interesting to the available knowledge from the literature. There are many papers describing the relationships between social status or cultural level or wealth or religious believes with adherence to **Young-Parent Communication?** What do the authors wish to add with their work? Is there any specific focus to be described within the specific area of interest in your study area? Please provide some extra reason for having specific interest in these findings. The abstract background particular is not focused and aim is not described enough. Please rephrase and detail more. Be focused please. The conclusion of the abstract is also not clear.

**Major**:
1. The Background is not well organized, and it missed some core parts that should have been addressed for a better justification of the current research. (1) First, the authors should tell us more about the importance of parent communication with their children in every aspects of their life. Does the government have ever conducted any policy interventions targeting to solve that reproductive issue? If yes what are the interventions? Secondly why is it interesting and important to focus on maternal healthcare service in Ethiopia? I wish to know the contextual motivations underlying your research.

**Method, result, and discussion**

1. What are the justifications for including those covariates in the model
2. Have the authors done: multicollinearity test, sensitivity test, and robustness test? If so, please add the results as an endnote
3. Using cross-sectional data would inevitably face the issue of endogeneity. Have the authors taken the endogeneity issue into consideration? For example, can you use the IV method to make your research findings more robust and convincing?
4. Results
   Goodness of fit measures for estimated models should be provided in the regression table.
5. In the Discussion section, please give some detailed policy suggestions based on your findings.
